# General Parenting Styles and Children's Obesity Risk: Changing Focus

**DOI:** 10.3389/fpsyg.2018.02119

**Published:** 2018-11-06

**Authors:** Junilla K. Larsen, Ester F. C. Sleddens, Jacqueline M. Vink, Jennifer O. Fisher, Stef P. J. Kremers

**Affiliations:** ^1^Behavioral Science Institute, Radboud University, Nijmegen, Netherlands; ^2^Department of Health Promotion, School of Nutrition and Translational Research in Metabolism, Maastricht University Medical Center, Maastricht, Netherlands; ^3^Department of Social and Behavioral Sciences, Center for Obesity Research and Education, Temple University, Philadelphia, PA, United States

**Keywords:** food parenting practices, general parenting, child eating, child BMI, moderation effect

Increasing attention has been given to direct associations of general parenting styles with children's obesity. General parenting styles (i.e., authoritarian, authoritative, permissive, and uninvolved) refer to the broad emotional context reflecting childrearing across situations and domains (Darling and Steinberg, 1993). Parenting styles focus less on what parents do (i.e., behavior-specific parenting practices) and more on how they do it in general (Power, 2013). In this commentary, we argue that general parenting styles should be conceptualized as a contextual factor that may moderate the influence of weight-related (e.g., food) parenting practices on behavior and weight outcomes among children, as opposed to having direct effects on those outcomes.

## Arguments against a direct link between general parenting styles and child obesity

First, the co-occurrence of secular *positive* trends toward authoritative parenting and childhood obesity appear to be in contrast with a direct *negative* link between authoritative parenting and children's obesity reported (Sokol et al., 2017). There has been a long-term decline in authoritarian parenting (i.e., high control; low warmth) and movement toward more authoritative (i.e., high control; high warmth) child rearing practices (Campbell and Gilmore, 2007; Zervides and Knowles, 2007; Doepke and Zilibotti, 2017) during roughly the same time period child obesity rates have risen to epidemic proportions (NCD Risk Factor Collaboration (NCD-RisC)., 2017). Although intergenerational comparison is limited by generational changes in obesogenic environment and meaning of certain parenting styles, this positive co-occurrence of secular trends is remarkable and argues against a direct negative association.

Second, a systematic review suggests that “parenting style” is a risk factor for childhood obesity in higher, but not low, SES families, although future research is needed to clarify the influence of the different styles (Mech et al., 2016). If general parenting styles were to have a direct effect on child obesity rates, then one might expect this association to be present among populations with increased vulnerability to obesity, such as low SES groups. However, comparison is limited as most studies have been biased toward middle and high SES populations (Gicevic et al., 2016). Only three studies directly compared the association between general parenting styles and childhood obesity in different SES groups. One study among 8–10 year olds did not find moderation effects of primary caregiver's education level (Rodenburg et al., 2011). Two other studies found evidence for moderation. One of these studies computed SES from parental education and occupational status, included children around 7 years, and found that permissive parenting was significantly associated with child obesity only among higher SES groups (Topham et al., 2010). The power in this study may not have been sufficient to find significant moderation with authoritarian parenting. The other study was highly powered and found that, compared to authoritative parenting, authoritarian, or negligent parenting was associated with increased likelihood of obesity only among preschool children not living in poverty, while poverty was not a moderator among school-age children (Kakinami et al., 2015). This might suggest that associations of general parenting styles with especially *young* children's obesity (age 7 and below) do not exist in low SES groups. This is remarkable as parents are particularly important for younger children and low SES groups have increased vulnerability to young childhood obesity (Manios et al., 2018).

Finally, most studies providing evidence in favor of a link between general parenting styles and children's obesity have not accounted for a number of potentially important confounders (Sokol et al., 2017). Consequently, it is not possible to rule out influences of unmeasured “third variables,” such as nutrition knowledge, parental stress, or access to grocery stores. To conclude, we suggest that evidence to date does not support a direct link between general parenting styles and children's obesity. The absence of such a direct link, however, does not mean that general parenting styles are inconsequential (Cislak et al., 2012).

## Arguments to consider the importance of parenting context

Several strong theoretical frameworks have proposed that general parenting styles moderate the influence of specific parenting practices on the child (e.g., Darling and Steinberg, 1993; Wachs, 1999). Evidence from survey studies on food parenting practices, referring to food structure (e.g., food rules or modeling), coercive control (e.g., food restriction or pressure), or autonomy support (e.g., food encouragement or praise) (Vaughn et al., 2016), largely supports this moderating role of general parenting styles (Larsen et al., 2015). Rhee and colleagues found that observed baseline general parenting may influence a child's ability to lower weight during a standard family-based behavioral weight control program (Rhee et al., 2016). Moreover, childhood obesity intervention trials that address parenting styles besides lifestyle have been successful. However, only two intervention studies compared components (i.e., lifestyle versus parenting). These studies support the idea that combined effects of general parenting styles (i.e., stimulating “authoritative parenting”) and lifestyle components are more promising for reducing child obesity than an exclusive focus on general parenting styles. Small and moderate effect size differences were reported (see Gerards et al., 2011 for a review). Future research should compare components of exclusive lifestyle with combined lifestyle and parenting approaches to better distinguish moderation effects.

A moderating role of general parenting may be particularly important for understanding the extent to which parenting practices are experienced as more or less controlling depending on the broader context in which they occur (Patrick et al., 2013; Langer et al., 2017). Children whose parents have authoritative parenting styles may be more likely to conform to parental directives compared to parents having other styles. Additionally, general parenting styles may influence child self-control and internalizing symptoms (Moilanen et al., 2015; Pinquart, 2017), and these mechanisms may interact with food parenting practices (e.g., food availability, accessibility, and food rules), rather than having an effect on their own. Finally, it might also be that authoritative parents use more effective types of controlling practices than other parents and are more consistent where specific food parenting practices have greater impact because they are more frequently used (Larsen et al., 2015). More naturalistic designs [e.g., observational and ecological momentary assessment (EMA) studies] may yield greater understanding of the extent to which general parenting styles influence the impact of specific weight-related (e.g., food) parenting practices or, alternatively, are confounded by use of other practices (i.e., more effective forms) or the consistency with which certain practices are used.

Observational studies can provide unique insight on parenting by characterizing influences on subtle dimensions of children's weight-related (e.g., eating) behavior that are more proximal to parenting than weight status and not easily assessed in survey studies. For instance, Lucas-Thompson and colleagues focused on general parenting and found that observed limit setting (but not observed warmth or self-reported parenting style) was related to the healthfulness of food choices during grocery shopping (Lucas-Thompson et al., 2017). Moreover, Moens and colleagues observed a specific food parenting practice (i.e., encouragement) and found that this practice was more prevalent among parents of healthy-weight children compared to overweight children (Moens et al., 2018). To the best of our knowledge, there are no observational studies combining information on food or other weight-related parenting practices and general parenting. This is an important target for future research.

Moreover, EMA studies are important because they can distinguish between short-term fluctuations in weight-related parenting practices and the causes and consequences of these fluctuations. Research has shown that parenting can vary across time and context. A recent EMA study by Berge et al. (2018) revealed that interpersonal conflicts between parents and children were related to use of restrictive feeding practices at the subsequent evening meal. Notably, Berge and colleagues additionally found that transient stressors, such as these interpersonal conflicts, were associated with serving more fast food at meals (Berge et al., 2018), and these fast food meals could have induced greater parental use of food restriction. Inclusion of experimental parts (e.g., manipulate type of meal served) in EMA (and observational) studies may yield insight into causality. Notably, there are few studies that have employed EMA in the area of obesity, and exceptions have measured parents *or* children (Engel et al., 2016). An important target for future research is to combine EMA information from both parents and children, including information on (food) parenting practices and general parenting. EMA can be successfully implemented with children from age seven and older (Heron et al., 2017). Finally, problems with EMA compliance rates may be tackled by inclusion of personalized EMA prompts.

To conclude, this commentary provides argumentations for changing focus from evaluating direct links between general parenting and child obesity to conceptualizing general parenting style as a contextual factor and potential moderator of the impact of specific weight-related parenting practices on children's eating behavior and obesity risk. Future naturalistic studies should include measurements of children's behaviors, which are more proximal to parenting than weight status. Moreover, innovative and personalized EMA studies with experimental parts among both children and parents simultaneously may yield better understanding of the interactions between weight-related parenting practices and general parenting influences.

## Author contributions

JL drafted and shortened the paper. SK oversaw paper process and reviewed the paper. ES reviewed the paper and added literature and references. JV added critical arguments and reviewed the paper. JF reviewed and edited the paper.

### Conflict of interest statement

The authors declare that the research was conducted in the absence of any commercial or financial relationships that could be construed as a potential conflict of interest.

## References

[B1] Berge J. M. Tate A. Trofholz A. Fertig A. Crow S. Neumark-Sztainer D. Miner M. (2018). Examining within- and across-day relationships between transient and chronic stress and parent food-related parenting practices in a racially/ethnically diverse and immigrant population. Int. J. Behav. Nutr. Phys. Act. 15:7. 10.1186/s12966-017-0629-1 29338753 PMC5771034

[B2] Campbell J. Gilmore L. (2007). Intergenerational and continuities and discontinuities in parenting styles. Aust. J. Psychol. 59, 140–150. 10.1080/00049530701449471

[B3] Cislak A. Safron M. Pratt M. Gaspar T. Luszczynska A. (2012). Family-related predictors of body weight and weight-related behaviors among children and adolescents: a systematic umbrella review. Child Care Health Dev. 38, 321–331. 10.1111/j.1365-2214.2011.01285.x 21752064

[B4] Darling N. Steinberg I. (1993). Parenting style as context: an integrative model. Psychol. Bull. 113, 487–496.

[B5] Doepke M. Zilibotti F. (2017). Parenting with style: altruism and paternalism in intergenerational preference transmission. Econometrica 85, 1331–1371. 10.3386/w20214

[B6] Engel S. G. Crosby R. D. Thomas G. Bond D. Lavender J. M. Mason T.. (2016). Ecological Momentary Assessment in eating disorder and obesity research: a review of the recent literature. Curr. Psychiatry Rep. 18:37. 10.1007/s11920-016-0672-7 26893235

[B7] Gerards S. M. Sleddens E. F. Dagnelie P. C. de Vries N. K. Kremers S. P. (2011). Interventions addressing general parenting to prevent or treat childhood obesity. Int. J. Pediatr. Obes. 6, e28–e45. 10.3109/17477166.2011.575147 21657977

[B8] Gicevic S. Aftosmes-Tobio A. Manganello J. A. Ganter C. Simon C. L. Newlan S.. (2016). Parenting and childhood obesity research: a quantitative content analysis of published research 2009-2015. Obes. Rev. 17, 724–734. 10.1111/obr.12416 27125603

[B9] Heron K. E. Everhart R. S. McHale S. M. Smyth J. M. (2017). Using mobile-technology-based Ecological Momentary Assessment (EMA) methods with youth: a systematic review and recommendations. J. Pediatr. Psychol. 42, 1087–1107. 10.1093/jpepsy/jsx078 28475765

[B10] Kakinami L. Barnett T. A. Séguin L. Paradis G. (2015). Parenting style and obesity risk in children. Prevent. Med. 75, 18–22. 10.1016/j.ypmed.2015.03.005 25797329

[B11] Langer S. L. Seburg E. JaKa M. M. Sherwoord N. E. Levy R. L. (2017). Predicting dietary intake among children classified as overweight or at risk for overweight: independent and interactive effects of parenting practices and styles. Appetite 1, 72–79. 10.1016/j.appet.2016.12.011 27940314 PMC5259556

[B12] Larsen J. K. Hermans R. C. J. Sleddens E. F. C. Engels R. C. M. E. Fisher J. O. Kremers S. P. J. (2015). How parental dietary behavior and food parenting practices affect children's dietary behavior. Interacting sources of influence? Appetite 89, 246–257. 10.1016/j.appet.2015.02.012 25681294

[B13] Lucas-Thompson R. G. Graham D. J. Ullrich E. MacPhee D. (2017). General and food-selection specific parenting style in relation to the healthfulness of parent-child choices while grocery shopping. Appetite 108, 353–360. 10.1016/j.appet.2016.10.019 27756635

[B14] Manios Y. Androutsos O. Lambrinou C. P. Cardon G. Lindstrom J. Annemans L. (2018). A school- and community-based intervention to promote healthy lifestyle and prevent type 2 diabetes in vulnerable families across Europe: design and implementation of the Feel4Diabetes-study. Public Health Nutr. 12, 1–10. 10.1017/S1368980018002136 PMC10260800 30207513

[B15] Mech P. Hooley M. Skouteris H. Williams J. (2016). Parent-related mechanisms underlying the social gradient of childhood overweight and obesity: a systematic review. Child Care Health Dev. 42, 603–624. 10.1111/cch.12356 27316858

[B16] Moens E. Goossens L. Verbeken S. Vandeweghe L. Braet C. (2018). Parental feeding behavior in relation to children's tasting behavior: an observational study. Appetite 120, 205–211. 10.1016/j.appet.2017.08.028 28864254

[B17] Moilanen K. L. Rasmussen K. E. Padilla-Walker L. M. (2015). Bidirectional associations between self-regulation and parenting atyles in early adolescence. J. Res. Adolesc. 25, 246–262. 10.1111/jora.12125

[B18] NCD Risk Factor Collaboration (NCD-RisC) (2017). Worldwide trends in body-mass index, underweight, overweight, and obesity from 1975 to 2016: a pooled analysis of 2416 population-based measurement studies in 128·9 million children, adolescents, and adults. Lancet 390, 2627–2642. 10.1016/S0140-6736(17)32129-3 29029897 PMC5735219

[B19] Patrick H. Hennessy E. McSpadden K. Oh A. (2013). Parenting styles and practices in children's obesogenic behaviors: scientific gaps and future research directions. Childhood Obes. 9, 73–86. 10.1089/chi.2013.0039 23944926 PMC3746290

[B20] Pinquart M. (2017). Associations of parenting dimensions and styles with internalizing problems of children and adolescents: an update meta-analysis. Dev. Psychol. 53, 873–932. 10.1037/dev0000295 28459276

[B21] Power T. G. (2013). Parenting dimensions and styles: a brief history and recommendations for future research. Childhood Obes. 9, S14–S21. 10.1089/chi.2013.0034 23944920 PMC3746212

[B22] Rhee K. E. Jelalian E. Boutelle K. Dickstein S. Seifer R. Wing R. (2016). Warm parenting associated with decreasing or stable child BMI during treatment. Childhood Obes. 12, 94–102. 10.1089/chi.2015.0127 26895374 PMC4817557

[B23] Rodenburg G. Kremers S. P. J. Oenema A. Van de Mheen D. (2011). Psychological control by parents is associated with a higher child weight. Int. J. Pediatr. Obes. 6, 442–449. 10.3109/17477166.2011.590203 21780869

[B24] Sokol R. L. Qin B. Poti J. M. (2017). Parenting styles and body mass index: a systematic review of prospective studies among children. Obes. Rev. 18, 281–292. 10.1111/obr.12497 28086262 PMC5436909

[B25] Topham G. L. Page M. C. Hubbs-Tait L. Rutledge J. M. Kennedy T. S. Shriver L.. (2010). Maternal depression and socio-economic status moderate the parenting style/child obesity association. Public Health Nutr. 13, 1237–1244. 10.1017/S1368980009992163 19968899

[B26] Vaughn A. E. Ward D. S. Fisher J. O. Faith M. S. Hughes S. O. Kremers S. P.. (2016). Fundamental constructs in food parenting practices: a content map to guide future research. Nutr. Rev. 74, 98–117. 10.1093/nutrit/nuv061 26724487 PMC4892304

[B27] Wachs T. D. (1999). Celebrating complexity: conceptualization and assessment of the environment, in Measuring Environment Across the Life Span: Emerging Methods and Concepts, eds Friedman S. L. Wachs T. D. (Washington DC: American Psychological Association), 357–92.

[B28] Zervides S. Knowles A. (2007). Generational changes in parenting styles and the effect of culture. J. Appl. Psychol. 3, 65–75. 10.7790/ejap.v3i1.81.7

